# A Contemporary Review of Surgical Options in Laryngotracheal Stenosis

**DOI:** 10.1007/s12070-024-05209-2

**Published:** 2024-11-15

**Authors:** Julia Śladowska, Anna Rzepakowska

**Affiliations:** 1 Students Scientific Research Group, Department of Otorhinolaryngology Head and Neck Surgery, Medical University of Warsaw, Warsaw, Poland; 2 Department of Otorhinolaryngology, Head and Neck Surgery, Medical University of Warsaw, 1a Banacha Str., Warsaw, 02-097 Poland

**Keywords:** Airway stenosis, Glottic web, Subglottic stenosis, Posterior glottic stenosis

## Abstract

Upper airway stenosis is a potentially life-threatening condition that can occur at the level of the larynx, trachea, or multiple sites. It compromises breathing, coughing up secretions or voice production. To date, a wide range of endoscopic and open procedures have been described. Different techniques involve variable decannulation times, recurrence rates or complications. Improved breathing is a major concern for each method. However, other laryngeal functions should also be maintained with optimal voice quality and swallowing function. All these features create a constant challenge for stenosis surgery and, consequently, the need to verify and improve existing methods. A literature review was performed to provide an overview of the different treatment methods utilized in particular localizations. Supraglottic stenosis has a high rate of recurrence; nevertheless, it has a better prognosis than glottic or subglottic stenosis does. In this location, endoscopic surgery is preferable because of its decreased morbidity and optimal wound healing. Glottic stenosis includes bilateral vocal fold palsy (BVFP) and anterior and posterior glottic stenosis. Endoscopic resection combined with readhesion-reducing treatment is recommended for less advanced scars. Endoscopic laterofixation is a safe and easy method to perform for BVFP when there is potential for functional recurrence. Posterior cordotomy with partial arytenoidectomy is selected for patients with persistent disease. Subglottic stenosis is graded according to the Cotton–Myer classification, and open surgical procedures are more successful than endoscopic procedures in terms of decannulation rates and disease-free recurrence periods. The current guidelines concerning the treatment of LTS are still heterogeneous and cover a wide spectrum of procedures. Consequently, the therapy should be tailored individually to the patient, and the operator should be familiar with advantages related to different approaches. Future protocol development requires the standardization of qualification criteria and the verification of respiratory, voice and swallowing results.

## Introduction

Upper airway stenosis is a potentially life-threatening condition that can occur at the level of the larynx (supraglottis, glottis, or subglottis), trachea, or multiple sites. If severe, it compromises breathing, coughing up secretions or voice production. It may result from different etiologies that can be divided into either congenital or acquired. Currently, the most common cause of laryngotracheal stenosis (LTS) is endotracheal intubation, whereas historically, infection (primarily syphilis, tuberculosis, typhus, or diphtheria) and external trauma have been the major factors. Other common etiologies include idiopathic (concerning Caucasian women with an unknown etiology), autoimmune, radiation, neoplasm and collagen-vascular diseases [1].

Although LTS is relatively common in otorhinolaryngology practice, management of the stenosed area still poses a major challenge. The estimated prevalence of the disease is thought to be 1:200 000 [2]. Nevertheless, the risk of developing postintubation LTS varies among studies, and it usually presents 4–6 weeks after onset.

To date, a wide range of endoscopic and open procedures have been described (Table 1). Different techniques involve different decannulation times, recurrence rates or complications. The comparison of these studies is difficult because of the use of diverse and often unspecified methodologies regarding the inclusion criteria as well as the varying accuracy of functional results assessment. Improved breathing is a major concern for each method. However, other laryngeal functions should be maintained with optimal voice quality and swallowing function. This creates a constant challenge for stenosis surgery and, consequently, the need to verify and improve existing methods. Consequently, to achieve a satisfying treatment goal, surgeons should be familiar with all techniques. For this reason, the aim of our study is to provide an overview of different treatment methods utilized in particular localizations that have been proposed in the literature. We believe that these findings could lead to the development of future therapeutic protocols.

**Table 1:** Review of surgical therapies for upper airway stenosis at different levels

		Endoscopic	Open
Supraglottic		• scar excision with CO_2_ laser with/without balloon dilation/stents/keels/adjuvant therapies [4, 5] • scar excision with KTP laser [3] • Z- plasty [6]	• scar excision via laryngofissure with stent placement [7] • hyothyroepiglottic laryngectomy [8]
Glottic	Vocal fold paralysis	• posterior (transverse) cordotomy [13] • arytenoidectomy [15] • endoscopic resection with lateralization [17–19] • endoscopic arytenoid abduction lateropexy [22] • microtrapdoor flap [32, 33]	• submucosal cordectomy/arytenoidectomy via laryngofissure [42, 43]
	Anterior glottic stenosis	• resection with keel/stent placement [24] • resection with mucosal graft/flap suturing [28] • microtrapdoor flap [33] • resection with MMC application [34, 35]	• resection with stent
	Posterior glottic stenosis	• resection with keel/stent placement [23] • resection with mucosal flap suturing [30, 31] • microtrapdoor flap [32, 33]	• resection with graft/stents [37–39] • scar excision via microthyrotomy [44]
Subglottic and tracheal		• scar excision with CO_2_ laser [49, 50] • balloon/rigid dilatation [52, 55] • Maddern procedure [56] • REACHER procedure [57] • stents [58, 59] • cryotherapy [60, 61] • resection with adjuvant therapies (IV steroids [65], MMC [62, 63], PPI [66], TMP-SMX [66], macrolides [66]) • microtrapdoor flap [32, 67]	• cricotracheal resection with anastomosis [53, 69] • cricoplasty [68] • segmental tracheal resection with end-to-end anastomosis [70–72] • slide tracheoplasty [73] • laryngotracheal reconstruction with expansion grafting [74]

## Supraglottic Stenosis

Supraglottic stenosis is very rare (approximately 3% of treated LTS) and has different causes, symptoms and treatment options from glottic and subglottic stenosis. It may involve the arytenoid cartilages, false vocal cords, aryepiglottic folds, or epiglottis. The majority of patients have coexisting dysphagia [3]. Supraglottic stenosis has a high rate of recurrence; nevertheless, it has a better prognosis than glottic or subglottic stenosis does. It is initially treated with tracheostomy and appropriate therapy for underlying disease [4].

Endoscopic surgeries are more favourable in the case of supraglottic stenosis because of their association with decreased morbidity and improved wound healing [4].

- **CO**_**2**_**(carbon dioxide)** lasers are the treatment of choice for supraglottic stenosis, as they improve wound healing in comparison with open procedures. It can be utilized in autoimmune disease, localized laryngeal stenosis, laryngeal tuberculosis or amyloidosis. Removal of scar tissue may be applied alone or in combination with balloon dilatation/adjuvant therapies/stents [4].
- **532-nm pulsed KTP (potassium titanyl phosphate) laser.** In this method, the contact mode KTP laser is used in the office setting to perform wedge resections at the region of the greatest scar volume, with the intervening tissue remaining intact. For larger scars, serial excisions are performed in separate areas of the stenosis to avoid circumferential trauma and further narrowing of the lumen. Stevens et al. reported that all his patients required a median of 2 treatments due to recurrent obstruction. Overall, the authors concluded that the KTP laser appears to be a more precise, less expensive and potentially safer alternative than the CO2 laser. Nevertheless, its use is currently limited to patients with stable, noncritical supraglottic stenosis [3].
- **Adjuvant therapies (mitomycin C (MMC)**, **steroids)**. MMC is an antineoplastic agent that inhibits RNA and protein synthesis and consequently reduces fibroblast proliferation. It has been reported that adjuvant therapies extend the time to restenosis and may even decrease the recurrence rate [4, 5].
- **Z-plasty.** In a series of nine adult patients, Yilmaz described a technically difficult but highly successful novel procedure. It involves incisions of mucosal triangles on the superior and inferior surfaces of the stenotic area, deep excision of scar tissue and covering the excised area by mucosal flap redraping. Follow-up (median was 4 years) did not reveal any restenosis. Nevertheless, it is a single-center, single-surgeon retrospective series, where bias is a risk [6].

**Open surgeries** are currently less common because of their association with increased morbidity due to chronic aspiration and poor wound healing [4].

- **Open surgery via laryngofissure.** The procedure includes laryngofissure with or without pharyngotomy for excision of the scar with the insertion of a stent. Although most patients reported by Kacker et al. needed postrepair endoscopic dilatation, the final success rate was 100%. The main conclusion of this series was that supraglottic stenosis is best treated with excision of the scar tissue followed by the use of soft endotracheal tube stents instead of laryngeal keels [7].
- **Open supraglottic laryngectomy.** Historically, it was the treatment of choice, but currently, open supraglottic laryngectomy is indicated only for severe cases. Minni et al. were the first to describe this method in 3 patients. The hyothyroepiglottic laryngectomy involves incision and exposure of the stenosis, resection of the hyoid bone, resection of the thyroid cartilage and lesion removal. The preservation of one mobile arytenoid guarantees swallowing function, and a 5-year follow-up revealed good phonation and respiratory function in all 3 patients [8].

### Glottic Stenosis

Glottic stenosis can be divided according to the etiology of neurologic origin, with bilateral vocal fold paralysis (BVFP) or glottic scarring with anterior (AGS), posterior (PGS) or complete stenosis.

- **BVFP** refers to the neurologic cause of vocal fold immobility. The incidence of BVFP is approximately one-third of all vocal fold paralysis cases. In contrast to patients with unilateral vocal fold paralysis, patients with BVFP usually present with airway compromise. The most common cause of BVFP is iatrogenic recurrent laryngeal nerve injury after thyroid surgery [9, 10].
- **AGS (anterior glottic stenosis)** in adult patients is most commonly caused by previous laryngeal surgical procedures. The characteristic symptoms are hoarseness and the inability to speak loudly. AGS can be seen in one of two forms: (1) anterior glottic web, when the scar tissue between true vocal cords is covered with epithelium and usually involves the anterior commissure, and (2) more complex, when true vocal cords, false vocal cords and ventricles are adherent to one another (vertical involvement).

The extent of the anterior glottic web can be categorized according to Cohen’s classification, which measures the length of the web in proportion to the glottis [11]:

•Grade I, ranging from 0 to 35% obstruction.

•Grade II, ranging from 35 to 50% obstruction.

•Grade III, ranging from 51 to 75% obstruction.

•Grade IV, ranging from 76 to 100% obstruction.

- **PGS (posterior glottic stenosis)** limits vocal fold abduction without concomitant dysphonia. It can be assessed via the Bogdasarian and Olson classification [12]:

•type I - interarytenoid scar, which does not extend to the posterior commissure (between the arytenoids only),

•type II - interarytenoid scar extending to the posterior commissure,

•type III - posterior commissure scar involving one cricoarytenoid joint,

•and type IV - posterior commissure scar involving both cricoarytenoid joints.

•Nevertheless, the most important prognostic factor in PGS is the mobility of the cricoarytenoid joints. Endolaryngeal procedures are warranted when the mobility of cricoarytenoid joints cannot be restored.

- **Complete glottic stenosis** is usually the result of inadequately managed external trauma. Nonetheless, infection or trauma from endolaryngeal surgery or prolonged intubation may worsen stenosis.

**Endoscopic surgeries** offer several benefits over open procedures, such as shorter hospitalization or a decreased risk of wound infection. They are most useful in the simplest (type I) and most severe (type IV) forms of PGS. Endoscopic resections of adhesive scar tissue can be easily performed with cold instruments or CO_2_ lasers; nevertheless, owing to the high risk of web reformation, they should be combined with readhesion-reducing treatment.

**Fig. 1:**
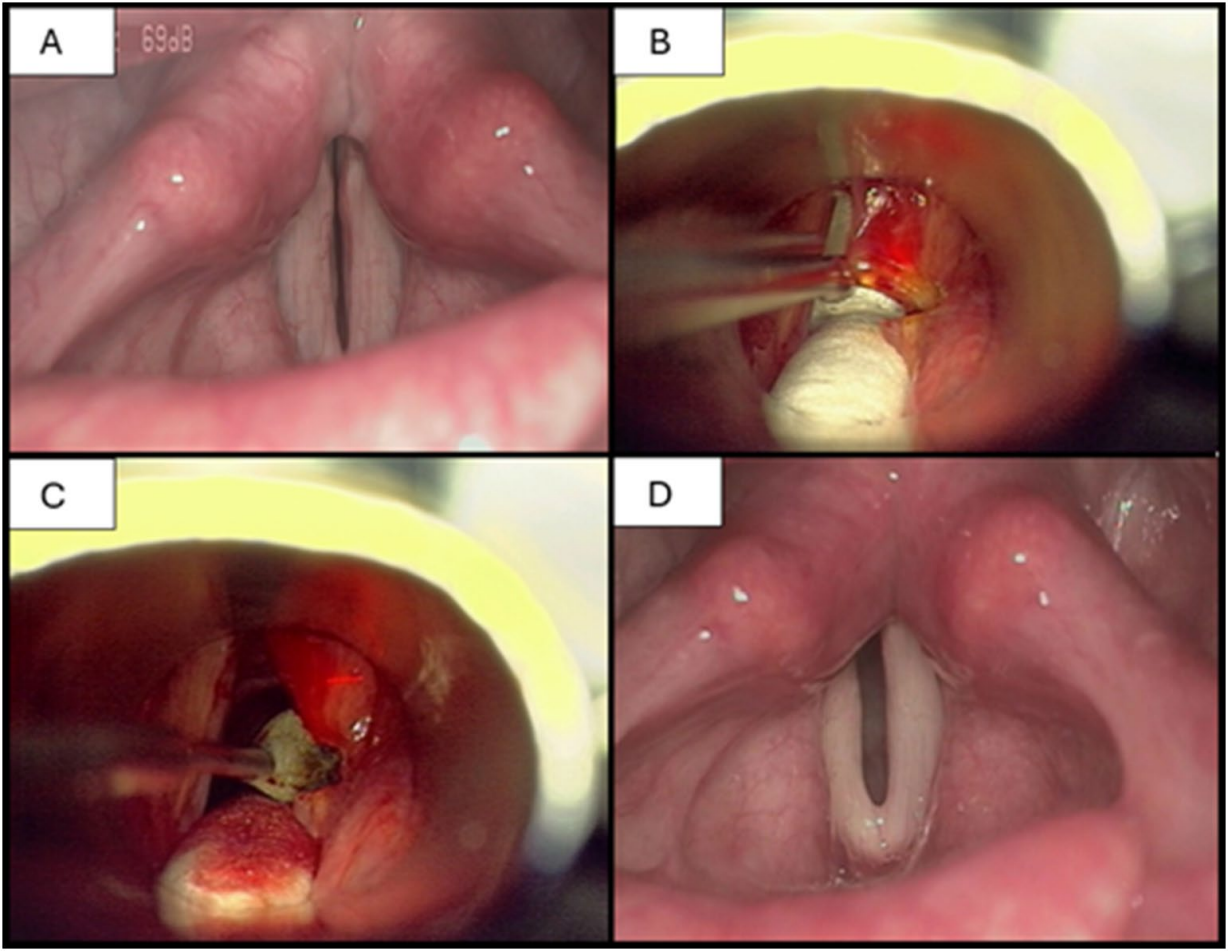
Transoral CO_2_ laser posterior cordotomy in bilateral vocal fold palsy

- **Endoscopic posterior (transverse) cordotomy.** It includes a transverse incision through the true vocal cord, anterior to the vocal process of the arytenoid, which should extend to the inner surface of the thyroid cartilage. It can be performed unilaterally or bilaterally. Laccourreye et al. revealed that the likelihood of successful one-step restoration of the airway is significantly increased during bilateral operations [13]. Bosley et al. reviewed the results of medial arytenoidectomy and transverse cordotomy for BVFP. Postoperative airway status, voice quality and perioperative complications were compared. He concluded that both methods are good treatment options for BVFP, with a low incidence of adverse events. Nevertheless, the limitation of this study is the small number of patients (11 patients treated with transverse cordotomy and 6 treated with medial arytenoidectomy) [10]. The advantage of posterior cordotomy is certainly the length of the procedure, averaging 10 min. Consequently, posterior cordotomy should be considered when the general condition requires the shortest amount of anaesthesia possible [14].
- **Endoscopic arytenoidectomy.** This is primarily described in the treatment of bilateral vocal fold immobility caused by recurrent nerve injury or cricoarytenoid ankylosis. However, its effectiveness was also confirmed in bilateral vocal cord midline fixation caused by cricoarytenoid fixation [14, 15]. While partial (medial) arytenoidectomy must be performed with a laser, for total arytenoidectomy, cold instruments can be utilized [16]. The main drawback of total arytenoidectomy is the high risk of postoperative aspiration and voice impairment. For this reason, the most commonly used method recently was endoscopic partial arytenoidectomy with partial/transverse cordotomy (Fig. 1). However, Yilmaz et al. concluded that if we preserve the arytenoid mucosa and suture it posterolateral rather than burn it with a CO_2_ laser, there is no significant difference in swallowing or voice parameters after endoscopic total or partial arytenoidectomy. Nonetheless, the authors indicate several disadvantages of total arytenoidectomy, such as the loss of arytenoid height, which may cause deglutition of the hypopharyngeal contents, or, less likely, the success of the revision operation. Conversely, total arytenoidectomy is 10 min shorter, making this procedure more feasible in patients with comorbidities, for whom the duration of the operation is an issue [16].

- **Endoscopic resection with lateralization.** The treatment options for BVFP with subsequent suture are classified by Lichtenberg as follows: reversible endoextralaryngeal lateralization (REExL), endoextralaryngeal laryngomicrosurgical lateralization (EExLL) and endoextralaryngeal laryngomicrosurgical lateralization with arytenoidectomy (EExLL with aryt.) [17]. REExL is indicated for patients with temporal immobility of the vocal folds, and it is suggested that the efficiency of this method (avoidance of tracheostomy) is best within 8 weeks when no fibrosis or ankylosis has developed. This procedure provides an adequate airway, although it does not restore vocal cord function. The vocal cords can be lateralized as long as they are nonfunctional [17, 18]. EExLL is reserved for patients with VFP but without cricoarytenoid joint ankylosis, whereas EExLL with aryt is reserved for patients with VFP and indicated when there is ankylosis. The advantages of these techniques include the ability to treat patients without tracheotomy and glottic mucosal preservation. The main disadvantage is increased breathiness of the voice [17, 19]. Endoscopic laterixation is also a safe and easy method to perform in children with BVFP [20].
- **Endoscopic arytenoid abduction lateropexy (EAAL).** Minimally invasive, reversible intervention for the treatment of BVFP, which allows for arytenoid stabilization in its maximally abducted position through endoscopically inserted sutures, without any resections. Compared with other endoscopic techniques, morphometric cadaver studies have revealed that EAAL provides the largest posterior glottic configuration [21]. The 18-month follow-up of 61 patients who had undergone EAAL revealed adequate and stable airways in all patients, bilateral or unilateral motion recovery with acoustic parameters in the physiological ranges in 23 patients (37.7%) and no significant motion recovery in 12 patients (19.7%) [22]. No swallowing difficulties were detected, and only 2 patients required repeated EAAL. Overall, EAAL was found to be an appropriate quasidynamic surgical solution that does not interfere with potential neuroregeneration and is beneficial in both transient and permanent palsy [22].
- **Endoscopic resection with keel/stent placement.** The use of a flat keel allows the avoidance of both round stents and endotracheal tubes as well as early mobilization of vocal cords [21]. Teflon keel placement has been reported to be a useful method for the treatment of AGS, PGS and complete glottic stenosis when laser surgery is not feasible [23–25]. Langman et al. suggested that if the gap between the arytenoids is less than 2 mm, then keel insertion should be the most preferable method. With the use of an endoscopically placed keel, most patients can be extubated unless there is cricoarytenoid joint fixation or partial tracheal stenosis [23].
- **Endoscopic resection with mucosal graft or flap suturing.** Many authors have reported mucosal flap suturing methods for the management of AGS. A classically described technique involves cutting the laryngeal web along the free edge, cutting the upper and lower flaps on opposite sides, flapping up the lower flap, flapping down the upper flap and suturing them [26]. Cao et al. suggested that the suturing technique alone yields adequate results in type I AGS, whereas webs that are larger (grades II–IV) or extend to the supraglottic or subglottic area are best treated with both keel placement and flap-suturing. However, to prevent restenosis, the keel should remain in the airway for 2–3 weeks, which poses a risk of detachment/migration and airway compromise [27]. Lahav et al. proposed the use of an anterior subglottic mucosal flap (ASGMF), a one-stage, keel-free technique for AGS repair. In his opinion, it is suitable for a wide range of anterior glottic lesions, as long as they do not extend to the supraglottic or subglottic levels [28]. Mucosal grafts are also used to reconstruct the anterior glottis. In the procedure described by Hsiung et al., the graft is harvested from the buccal mucosa and secured with temporary silastic stents. Follow-up of patients treated with this method revealed improvements in voice and excellent graft survival [29].

The endoscopic postcricoid advancement flap (EPAF) was described primarily by Goldberg and then modified by Damrose and Beswick. Its goal is to improve vocal fold mobility in patients with type II or III PGS. In both techniques, a postcricoid advancement flap and a gap between separated arytenoids are created. The authors recommend complete resection of the interarytenoid muscle because of the scar’s tendency to infiltrate this muscle. The flap can then be sutured to the mucosa of the anterior/inferior/medial base of the arytenoid or to the created subperichondrial “trap door” flap in the mucosa of the posterior subglottis. The above procedures allow for the preservation of voice quality and swallowing function [30, 31].

- **Microtrap door flap.** It is a treatment method for glottic, subglottic or tracheal stenosis [32, 33]. The surgery involves microlaryngoscopic elevation of the mucosal flap, removal of submucosal tissues with a cold knife or laser and redraping of the mucosal flap on the deepithelialized surface [33]. In 2016, Yilmaz et al. published his experience in treating 34 glottic stenoses with the above technique. The etiology was mainly failed surgery for BVFP. One year postoperatively, 33 patients were dyspnoea free on exertion, and all patients who underwent tracheotomy were decannulated. The success of this procedure is based on a reduced risk of restenosis due to mucosa preservation. An additional advantage is the use of a totally endoscopic approach and the possibility of outpatient or one-or-two-day hospitalization [33].
- **Endoscopic resection with MMC application.** Roh and Yoon examined the effects of topical MMC application to prevent AGS reoccurrence [34, 35]. They treated 16 patients with topical 0.4 mg/mL MMC for 5 min after transoral microresection of glottic lesions involving the anterior commissure. Postoperatively, 4 patients experienced local recurrences, and acceptable small webs in the anterior glottis occurred in 5 patients. Voice quality is affected mainly by the extent of vocal fold resection and extensive scarring rather than by the MMC per se [35]. A study on rabbits showed that MMC can also be helpful in the management of PGS [36].

### Open Surgeries

**Fig. 2:**
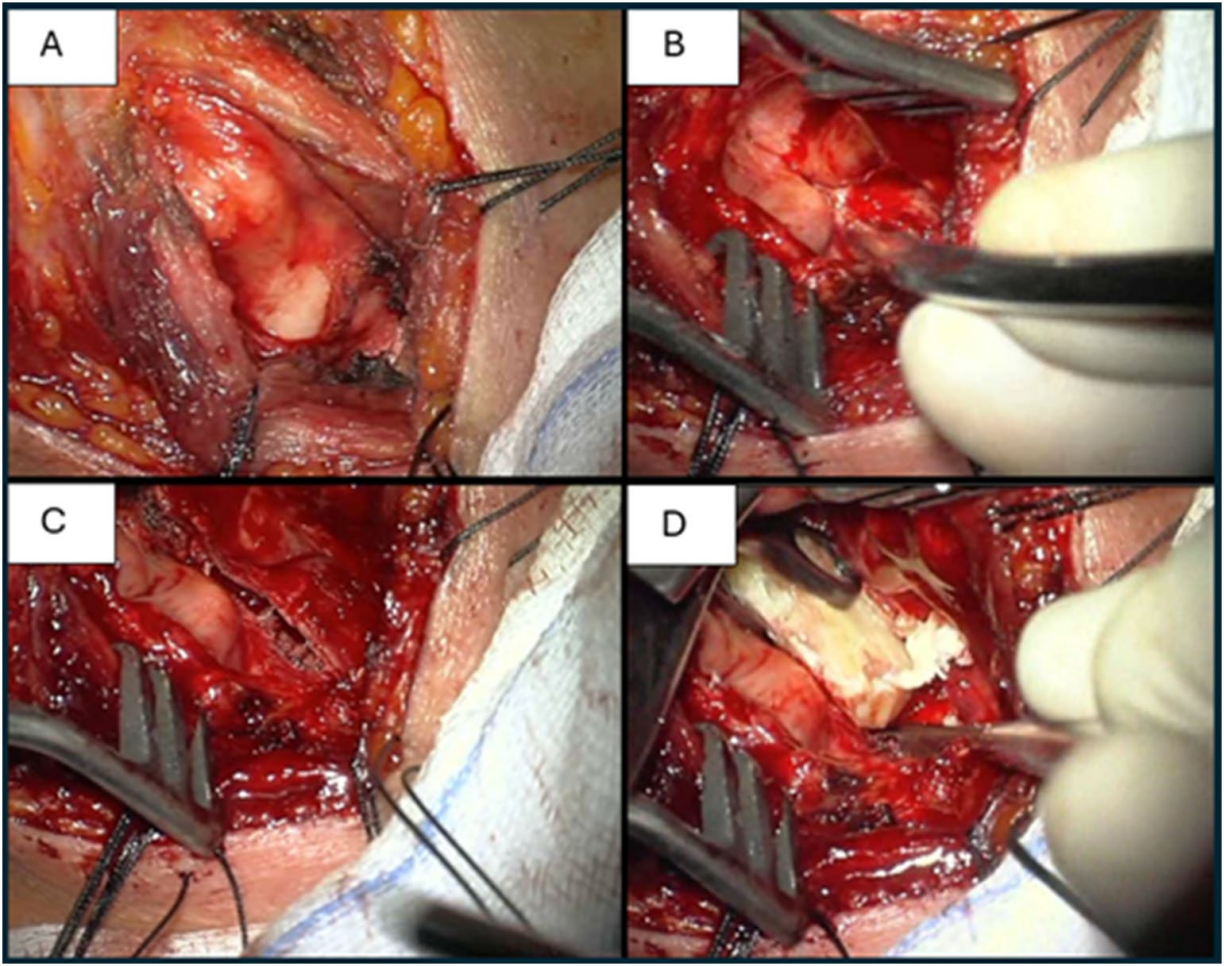
Laryngofissure with a rib cartilage graft in posterior glottic stenosis due to prolonged intubation (A thyroid cartilage exposure, B laryngofissure and cricoid cartilage plate exposure, C cricoid plate dissection, D insertion of a rib cartilage graft into the posteriorly dissected cricoid cartilage)

- **Open resection with grafting and stenting.** Resection via PGS involves laryngeal exposure, removal of scar tissue and placement of local mucosal flaps or grafting materials with or without stenting. A stent was left in place for 2–8 weeks. Owing to the technical difficulties associated with local mucosal flaps, free skin, buccal mucosa grafts or rib cartilages are commonly used (Fig. 2). Among these methods, decannulation success is high, and good voice quality is maintained [37–39]. In AGS, external procedures are indicated when the stenotic segment extends more than 5 mm subglotically [25]. Montgomery’s technique involves laryngofissure, resection of the stenotic segment, placement of a silicone keel between vocal cords and suturing it to the thyroid laminae. The keel can be removed in 2 weeks, with the tracheostomy remaining in place for 5 additional days [25, 40]. Laryngotracheoplasty allows for augmenting the cricoid lumen via an incision in conjunction with a rolled silastic stent, and it may be beneficial when the AGS is associated with cricoid abnormalities [25, 41].

- **Submucosal resection via the open laryngofissure approach.** Submucosal cordectomy and arytenoidectomy via laryngofissure are currently not recommended as the techniques of choice, but they can be employed when other procedures have been unsuccessful. The indications include BVFP and cricoarytenoid joint ankylosis. Both procedures are performed in the following steps: cross-section of the larynx at the vocal cord level, midline thyrotomy, mucosal flap elevation on the more affected side, muscle and scar tissue excision and placement of a stent, which is removed endoscopically in two weeks. Despite the fact that they are both effective in airway restoration, the voice quality remains poor in submucosal cordectomy [42, 43].
- **Minimally open approach resection.** The minicricothyrotomy approach is a minimally invasive external approach for the excision of the interarytenoid scar in patients with type I PGSs who cannot tolerate direct laryngoscopy. The excision is performed under the guidance of a transnasal or transoral fiberoptic laryngoscope. In the case report described by Mau et al., a 7-month follow-up revealed good voice quality, no aspiration, full abduction or adduction of the true vocal cords [44]. An approach involving an anterior window made in the thyroid cartilage (anterior laryngoplasty) has also been proposed, but in a cadaver study [45]. Kocak et al. reported the use of minicricothyrotomy in the management of high-pitched voices caused by glottic scarring secondary to laryngeal diphtheria infection in childhood [46].

### Subglottic and Tracheal Stenosis

Subglottic and tracheal stenosis is airway narrowing inferior to the glottis. The Cotton‒Myer grading system is widely utilized to determine the degree of stenosis by comparing the diameter of the endotracheal tube that can pass through the subglottic/tracheal lumen to the diameter of the expected age-appropriate endotracheal tube [47]:

- grade I − 0–50% occlusion.
- grade II − 51–70% occlusion.
- grade III − 71–99% occlusion.
- grade IV - no detectable lumen.

## Endoscopic Surgeries

In 2017, a systematic review including 834 patients was published, which revealed that open surgical procedures are more successful than endoscopic procedures in terms of decannulation success and the need for additional surgeries. Nevertheless, the literature lacks full descriptions of patients’ comorbidities, thus disorientating whether endoscopic surgeries are carried out in patients with greater morbidity or are actually less effective [48]. Endoscopic management can be used as a first treatment modality in a selected group of patients (grade I, some grade II and mild grade III, length of stenosis < 1.5 cm), as it is less invasive and risky, but it should not be repeated if the initial surgery fails [49].

- **CO**_**2**_**laser excision.** Monnier et al. determined that the laser should be used in ultrapulse mode with a fluence of 150 mJ/cm^2^ at a frequency of 10 Hz to avoid charring and heat diffusion into the surrounding tissue. The use of a CO_2_ laser to vaporize cartilaginous structures is contraindicated because of the risk of weakening the laryngotracheal framework and inducing restenosis [46]. In 2020, Ekbom et al. published his institution’s 20 years of experience with CO_2_ laser wedge excision in idiopathic subglottic stenosis (iSGS). This technique involves excision of large edges of stenotic tissue with preservation of small bridges to allow for remucosalization and prevention of circumferential scarring. The results revealed its superiority in decreasing the recurrence rate in comparison with endoscopic balloon dilatation and improving voice quality compared with cricotracheal resection and endoscopic dilatation [50].
- **Endoscopic dilatation (ED) with balloons or rigid dilators.** ED is the most common surgical approach in iSGS, which is typically soft. It is less commonly performed on harder, established stenoses. The technique can be safely applied in both adults and children, and it involves an endoscopic high-pressure balloon catheter, which is inflated at the level of the stenosis. The balloon pressure is maintained for 30 s or until the patient’s oxygen saturation drops below 92%. The procedure can be repeated several times with a single instance of general anaesthesia. After dilatation, the balloon is deflated and withdrawn [51, 52]. Among the three most popular approaches for iSGS (ED, cricotracheal resection, and endoscopic resection with adjuvant therapy (ERMT)), ED is associated with the highest risk of recurrence and lower voice outcomes than ERMT is [53]. In a pediatric population, the risk of dilation failure was shown to be associated with gastroesophageal reflux [51]. Another type of dilatation is rigid dilatation, which involves any solid instrument (bougie, bronchoscope, tracheal dilators) used to manually widen the airway. In a systematic review that compared balloon and rigid dilatation as primary therapies for LTS in the pediatric population, the authors concluded that rigid dilatation is successful for grade I and grade II/III subglottic stenosis, whereas balloon dilatation is recommended only for grade II/III subglottic stenosis [54]. Both procedures seem to be effective and safe; however, balloon dilatation achieves better long-term results [54, 55].
- **Maddern (endoscopic laryngotracheoplasty) and the REACHER procedure.** It is a novel, minimally invasive technique first introduced by Sandhu et al. in 2015. The procedure is reserved for patients who have failed other methods of treating subglottic stenosis and is ideal for those with high posterior cricoid scars. It is technically challenging, as it requires endoscopic resection of the subglottic mucosa without disturbing the cartilaginous framework. Then, an endoluminal stent is customized to the size of the resected segment and secured with a split-thickness skin graft from the thigh or buccal grafts to avoid subsequent granulation tissue formation/restenosis. The stent is removed endoscopically two weeks later [56]. The described procedure is similar to the REACHER procedure, with the exception that the second procedure is performed via the external neck approach [57].
- **Stents** can be divided into short-term (< 6 weeks) or long-term (≥ 6 weeks). Short-term stenting is usually used to stabilize the graft after reconstruction surgery. The most common stents are Aboulker stents, silicone stents, Montgomery laryngeal stents, endotracheal tubes and laryngeal keels. Long-term stenting is used when the previous methods fail. The most common stents are Montgomery T-tubes and Aboulker tubes. The complications associated with stenting include airway blockage and inflammation [58]. A study in a pediatric population demonstrated that long-term stenting improved the successful decannulation rate [59].
- **Cryotherapy.** Initially, it was carried out by using contact probes, but now, benign tracheal stenoses can be treated by using spray cryotherapy performed through different techniques under endoscopic control. The techniques include (1) rigid bronchoscopy, (2) suspension laryngoscopy, (3) laryngeal mask anaesthesia, and (4) intubation with an endotracheal tube. The evidence on this method is limited; nevertheless, it appears to be safe and effective, with rates of complications ranging from 0 to 19.3% (hypotension, desaturation, bradycardia, pneumothorax). Moreover, in contrast to laser therapy, it can be applied in patients who require 100% oxygen therapy [60, 61].
- **Endoscopic resection with adjuvant therapies.** The adjuvant options include inhaled and intravenous steroids, MMCs, proton pump inhibitors (PPIs), trimethoprim/sulfamethoxazole (TMP-SMX) and macrolides. Although opinions on adjuvant therapies vary, it has been shown that topical MMC is safe and effective in postponing restenosis. The symptom-free period was assessed to be ≥ 1 year in most cases [62]. The number of applications is associated with delaying recurrence but not with a complete cure [63]. Endoscopic treatment with MMC is economical if only 1 in 17 patients does not require open surgery [64]. Serial intralesional steroid injections also improve surgery-free intervals up to 4.6–12 months [65]. On the other hand, inhaled corticosteroids are not recommended, as the amount actually deposited at the subglottis is uncertain. Additionally, patients with iSGS and gastroesophageal reflux may benefit from PPIs. TMP-SMX and macrolides eliminate the pathogenic upper airway microbiome and are considered helpful in iSGS [66].
- **Microtrap door flap.** The procedure is described in this article. Dedo and Sooy reported that 9 out of 10 patients who underwent this method for subglottic or tracheal stenoses achieved a treatment goal. Nevertheless, the success rate in children under 5 years of age is poor [32, 67].

**Open surgeries** may be applied as a first treatment modality in severe cases (grade II, III and IV, length of stenosis > 1.5 cm, when the stenosis is cartilaginous or there is a loss of cartilage support) [49].

- **Cricotracheal resection (CTR) with anastomosis.** During the procedure, the cricoid cartilage and upper tracheal stenotic rings are incised vertically. The cricoid cartilage is subsequently resected anteriorly to the cricothyroid joints to expose the posterior cricoid plate, which is thinned to ~ 40% of its thickness. The upper trachea is subsequently separated from the esophagus, and the stenotic segment is resected. Finally, anterior thyrotracheal and lateral cricotracheal anastomoses are carried out. Patients with very high stenosis and lateral narrowing may benefit from tailored cricoplasty, which is a modification of the CTR [68]. Among other procedures, CTR has the most lasting effect but also has the greatest perioperative risk and decreased voice outcomes [53]. Complications include anastomotic difficulties such as granulations or subcutaneous emphysema and nonanastomotic difficulties such as wound infections or recurrent nerve palsies [53, 69]. For iSGS, the risk factors for anastomotic complications appear to be ANA negativity and postoperative edema [69].
- **Segmental tracheal surgery with end-to-end anastomosis.** The procedure is performed in the resection of fewer than six tracheal rings because resections over 4 cm are associated with an increased risk of failure [70]. Indications include a reduction in transverse tracheal diameter of 50–70% with clinical symptoms such as stridor or stress dyspnea [71]. Conventionally, in tracheal resection with end‒end anastomosis, surgical exposure of the larynx and trachea is achieved via a collar incision (eventually additional partial sternotomy when the stenosis is located distally). In this method, the trachea is dissected anterolaterally above and below the stenotic segment, and then, the incision progresses proximally and distally until normal tracheal rings are reached. The membranous part of the trachea is subsequently separated from the esophagus, the trachea is resected, and end-to-end anastomosis is performed. The operation is very successful. In a review of 901 patients who underwent segmental tracheal resection, 96% had satisfying airway results. The above study also revealed that anastomotic complications are related to diabetes, reoperation, young age (pediatric patients), BMI > 35 kg/m^2^ and the need for tracheostomy before surgery. Although some of them are unmodifiable, we should consider them carefully when stratifying the risk of the operation [70]. Postoperative complications vary among studies and include airway obstruction, wound infection, dysphagia, and inferior laryngeal nerve paralysis [72].
- **Slide tracheoplasty.** The technique is applied for cases of long-segment tracheal stenoses or complete tracheal rings (birth defects). In this surgery, the trachea is divided across the middle of the stenosis, the back of the lower tracheal segment and the front of the upper tracheal segment are cut, and the open ends are slid onto each other and sutured. As a result, an airway that is twice as wide and half as long is created. Although the procedure is very popular in the pediatric population, there are very few descriptions of its use in adults. Redmann et al. suggested that slide tracheoplasty can be performed in adults as a primary and revision surgery, with an overall success rate of 95%. However, more research is needed for further conclusions [73].
- **Laryngotracheal reconstruction with expansion grafting.** The procedure (Fig. 2) described previously in the article for the treatment of PGS is performed when subglottic stenosis extends to the level of the vocal folds. In this case, subglottic resection does not provide adequate treatment because this type of stenosis requires not only the removal of scar tissue but also the enlargement of the airway lumen. The graft is most commonly harvested from fifth or sixth rib cartilage with preservation of both sides of the perichondrium [74].

**Fig. 3:**
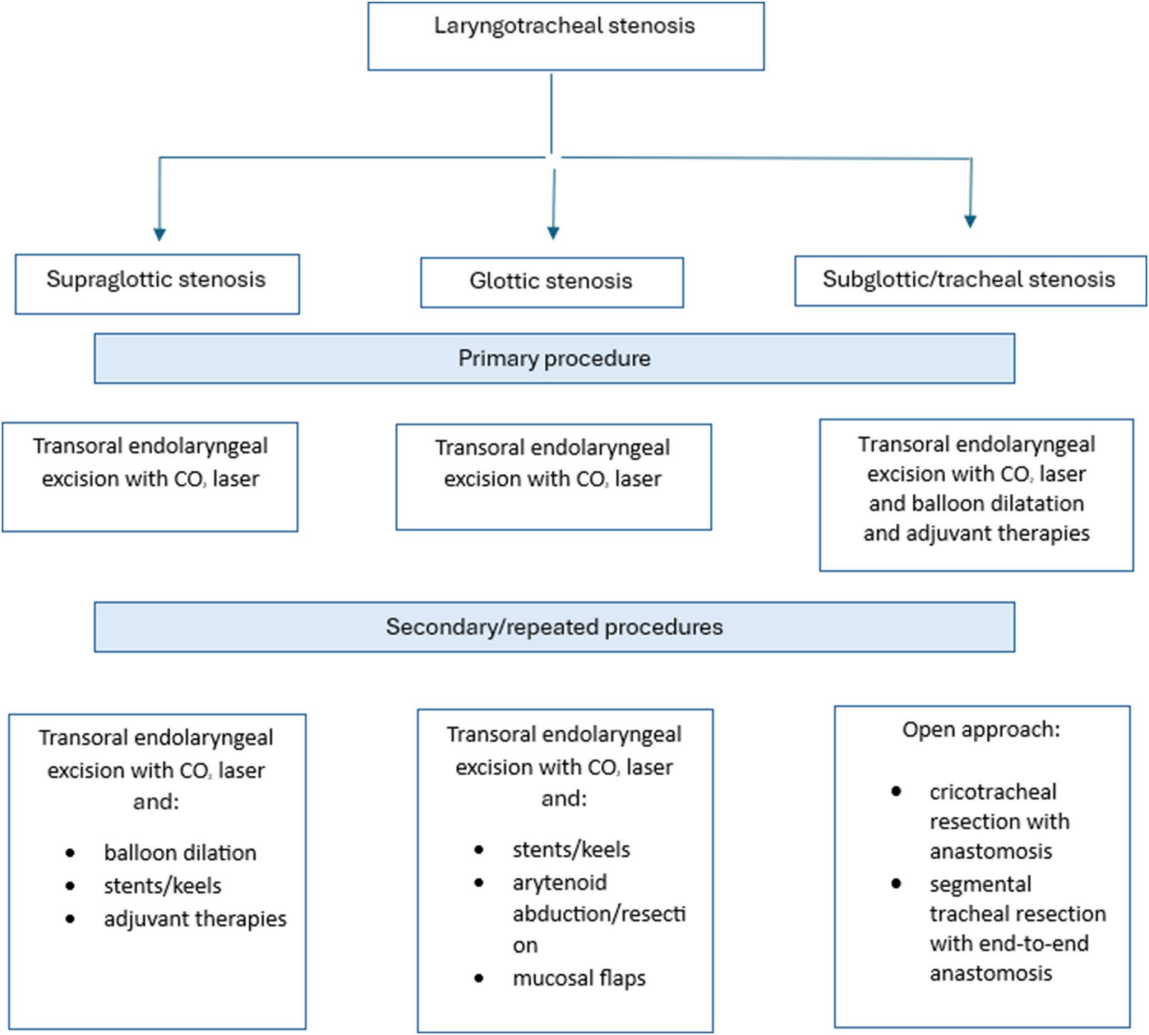
Algorithm for management of laryngotracheal stenosis

Summarizing the reviewed data on laryngotracheal stenosis management, we propose the following algorithm presented in Fig. 3.

## Conclusions

The current guidelines concerning the treatment of LTS are still heterogeneous and cover a wide spectrum of procedures. Owing to the relatively rare occurrence of stenosis and the significant consequences for the basic functions of breathing, phonation and swallowing, the cooperation of specialized centers is very important, and action is taken to develop treatment standards. Determining uniform criteria for inclusion in specific treatment modalities at individual stages of stenosis with the monitoring of breathing, voice and swallowing functions in a unified manner in the postoperative period will allow the development of optimal methods of management. At the same time, it is important to consider conducting research on the biological factors causing an individual predisposition of patients to progressive fibrosis and the formation of webs and stenosis in the upper respiratory tract.
